# Reproductive success delays moult phenology in a polar mammal

**DOI:** 10.1038/s41598-019-41635-x

**Published:** 2019-03-26

**Authors:** Roxanne S. Beltran, Amy L. Kirkham, Greg A. Breed, J. Ward Testa, Jennifer M. Burns

**Affiliations:** 1 Biology and Wildlife, University of Alaska Fairbanks, 2090 Koyukuk Drive, Fairbanks, Alaska 99775 USA; 2 Biological Sciences, University of Alaska Anchorage, 3101 Science Circle, Anchorage, Alaska 99508 USA; 3 Present Address: Ecology and Evolutionary Biology, University of California Santa Cruz, 115 McAllister Way, Santa Cruz, California 95060 USA; 4 College of Fisheries and Ocean Sciences, University of Alaska Fairbanks, 17101 Point Lena Loop Road, Juneau, Alaska 99801 USA; 5 Institute of Arctic Biology, University of Alaska Fairbanks, P.O. Box 757000, Fairbanks, Alaska 99775 USA; 6 Marine Mammal Laboratory, Alaska Fisheries Science Centre, National Marine Fisheries Service, National Oceanic and Atmospheric Administration, 7600 Sand Point Way N.E. F/AKC3, Seattle, W 98115 USA

## Abstract

Animals can respond to dynamic environments through phenological plasticity of life history events; however, changes in one part of the annual cycle can diminish the success of subsequent life history events. Our aims were to determine the associations between reproduction and moult phenology across years and to quantify phenological plasticity across varying environmental conditions. We conducted demographic surveys of 4,252 flipper-tagged Weddell seals (*Leptonychotes weddellii*) in the Ross Sea, Antarctica during four austral summers. At each sighting, seals were assigned a moult code based on the visible presence of new fur and the start date of each animal’s moult was back-calculated. Reproductive success and parturition dates were obtained for the breeding season prior to and following the moult. We found that successful reproduction delayed moult by 16 days relative to non-parturient females. Phenology of the intervening moult was indicative of previous reproductive dynamics but not predictive of subsequent reproductive outcomes. Across years, moult phenology varied by about two weeks and covaried strongly with sea ice break-out timing for all reproductive categories. Our findings suggest these polar mammals have some flexibility within the annual cycle that allows adjustment of moult phenology to fluctuating environmental conditions without compromising future reproductive success.

## Introduction

Animals can respond to dynamic environments through phenological plasticity of life history events^1,2^. However, changes in one part of the annual cycle can impact subsequent processes (i.e. carry-over effects^3,4^;) and these shifts can in turn diminish future success in foraging, breeding, or survival^5–7^. Among most vertebrates, it is uncommon for moult and reproduction to overlap due to the high energetic costs of both^8–10^. Because peak food availability^11,12^ and suitable climate^13^ are important to the success of both life history events^14,15^, an adaptive balance exists between breeding and moulting phenology^16^. Thus, it is important to study phenological variation in the larger context of annual cycles^17^ and survival^14,18,19^ in order for the ecological implications of dynamic environments and perturbations to be understood^20,21^.

Documenting moult progression is a prerequisite for understanding the carry-over effects of phenological disruptions; however, the moult is poorly understood relative to other life history events^22^. To date, the role of the moult as an intermediate life history event between two breeding seasons has been studied almost exclusively in birds^15,23^. In these studies, reproductive success has been found to delay moult relative to sexually mature but non-parturient conspecifics^13,14,24–27^. Given the apparent role of reproductive and stress hormones in delaying moult onset^28^, later reproduction and lower resource availability likely result in later moulting across birds and mammals.

In mammals, moult initiation and completion are difficult to identify because follicle growth precedes visible hair loss^29,30^ and the extent of hair loss can be difficult to determine. As a result, mammalian moult studies have been restricted to basic descriptions of where and approximately when moult occurs and how it influences animal behavior^31,32^. In seals (family *Phocidae*), some evidence suggests that moult is delayed in reproductively successful individuals^33–37^; however, the effects of reproduction on moult have primarily been established at the population level or in a limited number of captive animals. Thus, the drivers and consequences of moult duration and phenology in individuals are unclear.

In this study, we use free-ranging Weddell seals as a model species to evaluate the moult as an intermediate life history event between two reproduction events. Specifically, we address three aims: (1) to describe the duration and phenology of the moult across age, sex, reproductive categories, and environmental conditions; (2) to analyse the relationship between reproductive phenology (October-November, Year 1) and subsequent moulting phenology (January–March, Year 1) in paturient females; and (3) to understand the relationship between moulting phenology and reproductive outcomes in the following season (October-November, Year 2). As the most southern breeding mammal, Weddell seals have highly constrained annual cycles and serve as a useful model for understanding mammalian moult cycles. Further, the ages and reproductive histories of most individuals are known due to a 45-year demographic study^38,39^.

## Methods

Research activities were approved by National Marine Fisheries Service Marine Mammal permit #17411, University of Alaska IACUC protocols #419971 and #854089 and the Antarctic Conservation Act permit #2014-003 and were carried out in accordance with guidelines for handling marine mammals.

### Field methods

**Figure 1:**
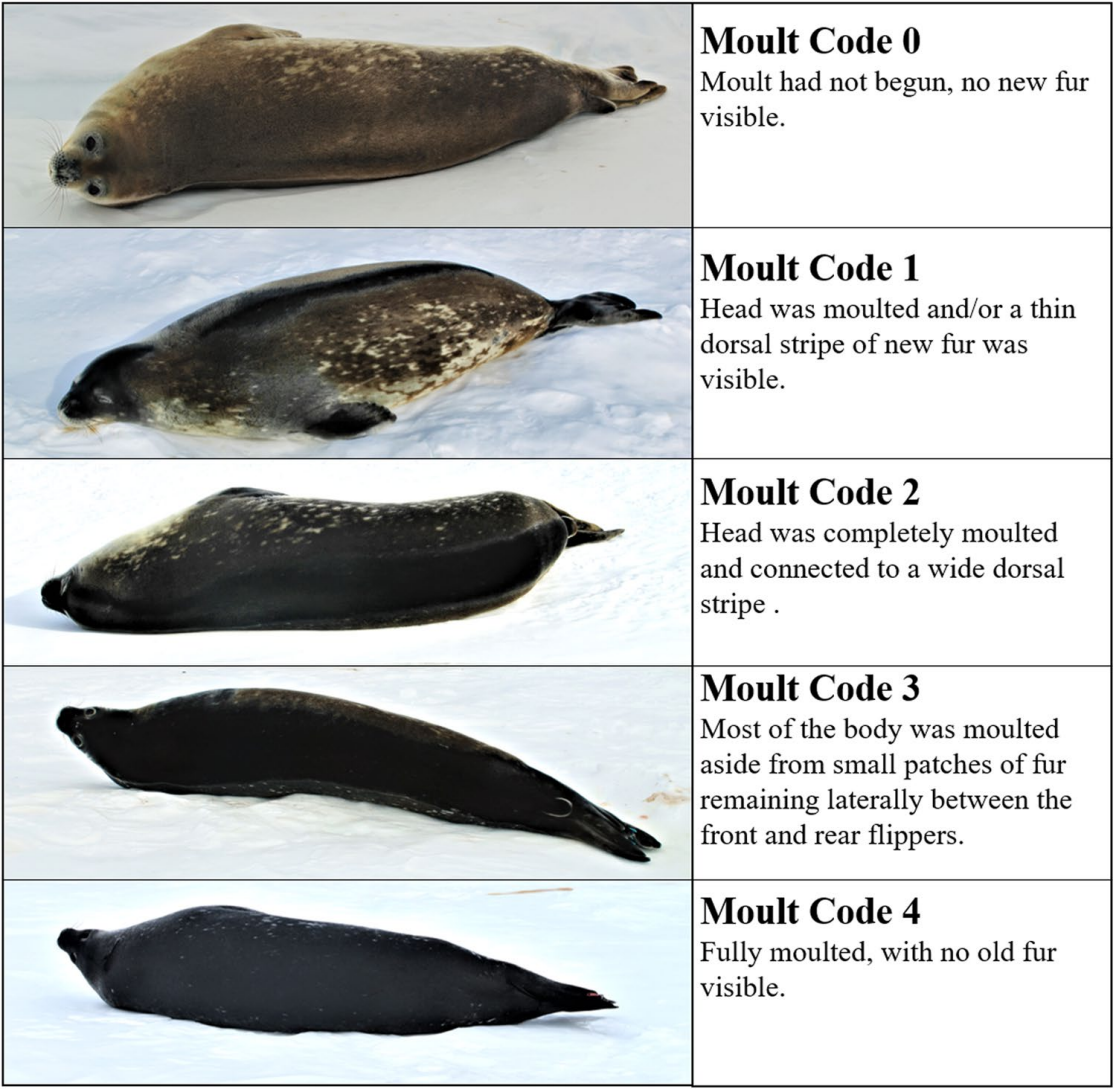
During surveys, each individual was assigned a moult code: 0 (unmoulted), 1 (head or dorsal stripe moulted), 2 (head and wide dorsal stripe moulted), 3 (flank starting to moult), or 4 (completely moulted).

In 2013–2017, we conducted semi-weekly surveys of Weddell seals (*Leptonychotes weddellii*) in Erebus Bay, Antarctica (77°S, 165°E). Each seal was approached and its flipper tag identification number, age class, and sex were recorded along with a qualitative moult code based on the visible presence of new fur (Fig. 1): code 0 - moult had not begun, no new fur visible; code 1- head moulted and/or a thin stripe of new fur visible along the spine; code 2 - head completely moulted and connected to a wide swath of new fur along the spine; code 3 - only small patches of unmoulted fur remained laterally between the front and rear flippers; and code 4 - fully moulted, no old fur visible. If the moult code could not be assigned because the animal was wet, covered in snow, or laying so that dorsal pelage was not visible, moult state was noted as unknown. Ages and sexes were obtained for tagged individuals based on a long-term demographic study^38–40^. Year is given as the austral summer each seal was observed moulting (e.g., 2013 is the 2013–14 austral summer season, including October 2013–December 2013 pupping and December 2013–March 2014 moulting) (Table 1). We use sea ice break-out date as a proxy for the timing of food availability because the annual ice break-out triggers a phytoplankton bloom that enhances productivity for seals via trophic linkages^41^. For each year, ice break-out date was obtained from satellite imagery using methods described in Beltran^42^.

**Table 1:** Information about moult surveys during 2013–2016, including quartiles for pupping dates and mean ± standard deviation moult initiation dates for each year and reproductive category.

	2013	2014	2015	2016
**Sighting Metadata**				
# Sightings	1810	1470	2212	3423
# Seals	1038	866	937	1411
# Survey Days	19	10	11	26
First Moult Survey Date	Jan 13	Jan 17	Jan 18	Jan 18
Last Moult Survey Date	Feb 13	Feb 14	Feb 15	Mar 8
**Pupping Quartiles**				
Minimum	*	Oct 11	Oct 13	Oct 14
25^th^ percentile	*	Oct 22	Oct 22	Oct 24
Median	Oct 29	Oct 26	Oct 27	Oct 28
75^th^ percentile	Nov 02	Oct 30	Oct 30	Nov 02
Maximum	Nov 15	Nov 26	Nov 13	Nov 19
**Moult Initiation Dates**				
Attendant Non-Parturient	Dec 28 ± 11.9^i^	Jan 05 ± 10.7^ii^	Jan 07 ± 9.9^ii^	Jan 12 ± 9.8^iii^
Non-Attendant Juvenile Females	Jan 06 ± 11.6^i^	Jan 07 ± 13.7^i^	Jan 11 ± 9.7^i^	Jan 17 ± 12.1^ii^
Attendant Juvenile Females	Jan 04 ± 11.3^i^	Jan 11 ± 6.7^ii^	Jan 15 ± 8.3^ii^	Jan 15 ± 14.6^ii^
Non-Attendant Adult Females	Jan 07 ± 14.5^i^	Jan 11 ± 11.8^i^	Jan 17 ± 11.7^i^	Jan 19 ± 9.6^i^
Attendant Parturient	Jan 19 ± 8.4^i^	Jan 15 ± 11.1^ii^	Jan 18 ± 9.2^i^	Jan 29 ± 13.2^iii^
Males	Jan 09 ± 13.1^i^	Jan 13 ± 10.6^ii^	Jan 16 ± 8.1^ii^	Jan 24 ± 12.9^iii^
Ice break-out date**	Jan 14	Jan 14	Jan 02	Feb 04
Colony attendance***				
Attendant Non-Parturient	22%	22%	18%	25%
Non-Attendant Parous	10%	6%	22%	20%
Attendant Parturient	68%	72%	60%	55%

Significant differences in moult initiation dates among years (by row, within a reproductive category) are denoted with Roman numerals (α level = 0.05).

*In 2013, the United States government shut down delayed the Weddell seal research program by several weeks, and pup tagging began only after 50% of the pups were born; as a result, the dates of the earlier quartiles are not known (see methods).

**Date when 7-day running mean of ice concentration falls below 50%. See Beltran^42^ for details.

***Colony attendance proportions for the following year.

**Figure 2:**
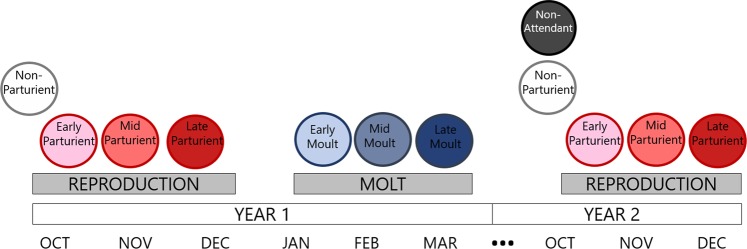
Based on individual sightings, adult female Weddell seals were assigned a phenology category for three life history events: reproduction in Year 1, moulting in Year 1, and reproduction in Year 2. Early, mid, and late correspond to the distribution 0–25%ile, 25–75%ile, and 75–100%ile, respectively; Non-Parturient had previously pupped but were not parturient in a given year; and Non-Attendant had been seen during the Year 1 pupping and moulting season but were not seen during the Year 2 pupping season.

Adult females were assigned a reproductive category for the pupping period preceding the moult season in which they were observed (October–December, hereafter Year 1 pupping period) and the pupping period following the moult (hereafter Year 2 pupping period). If adult females were observed with a dependent pup, they were considered to have pupped successfully (hereafter, attendant parturients). Each colony was visited every two to three days throughout the reproductive season, which allowed pupping success and precise birth dates of new-born pups (those with visible umbilical cord stumps) to be determined for many adult females per year. Using the pupping date distribution for new-borns to estimate the quantiles of pupping dates for each year, we categorized attendant parturient females as Early-, Mid-, or Late-Parturients (Table 2, Fig. 2). Alternatively, we categorized females who did not give birth (Attendant Juvenile Females, Attendant Non-Parturients) or who were not observed during the reproductive season (Non-Attendant Adult Females, Non-Attendant Juvenile Females). Because male breeding behavior is difficult to assess, we did not attempt to link breeding and moulting phenology in males. Due to logistical constraints in 2013, pupping quartiles calculations were modified slightly (see Supplemental Material).

**Table 2:** Details of reproductive categories assigned to each female during the period preceding each moult.

Reproductive Category	Gave birth?	Attended colony?	Details
Early-Parturient	Y	Y	Gave birth before the 25^th^ percentile of the pupping distribution.
Mid-Parturient	Y	Y	Gave birth between (or on) the 25^th^ and 75^th^ percentile of the pupping distribution.
Late-Parturient	Y	Y	Gave birth after the 75^th^ percentile of the pupping distribution.
Attendant Juvenile Female	N	Y	Seen during the breeding season without a pup and never recorded with a pup in any previous year (i.e., no pups produced yet in life).
Attendant Non-Parturient	N	Y	Seen during the breeding season without a pup but recorded with a pup in any previous year.
Non-Attendant Adult Female	N*	N	Not seen during the breeding season but recorded with a pup in previous years.
Non-Attendant Juvenile Female	N*	N	Not seen during the breeding season and never recorded with a pup in previous years.

*Given that there are no known pupping colonies near our study site, we assume that Non-Attendant individuals skipped pupping and temporarily emigrated, as described by Chambert, *et al*.^88^.

### Analytical methods

#### Estimating moult stage durations

**Figure 3:**
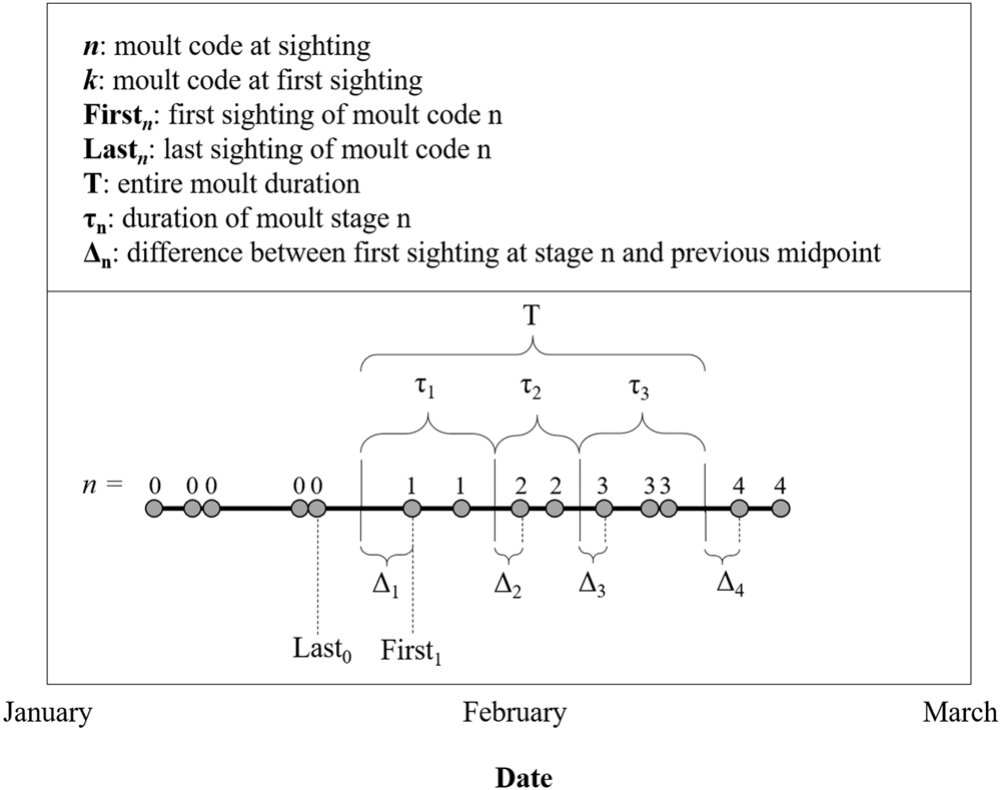
Moult sighting data from a theoretical animal with moult code *n* shown as numbers along the date axis. Each sighting is represented as a grey circle with moult code *n* shown. Mean moult stage durations τ_*n*_ were used to back-calculate a start date for each individual when the animal was not observed in a moult code *n*. A glossary of parameters is shown in the top panel.

Although an existing *R* package “moult”^43^ is available for analyses of moult dynamics, it does not allow for individual random effects in moult duration. Therefore, we developed custom functions in *R* (*R* Development Core Team 2017, version 3.3.2) to estimate the duration of moult based on subsequent sightings of a series of moult codes in each individual. Moult stage durations τ_*n*_ were calculated as the amount of time that passed between the observed moult stage (*n*) and the moult stages preceding (*n* − 1) and following (*n* + 1), using a midpoint approach (see Fig. 3). The moult codes 0, 1, 2, 3, and 4 represent each of the five stages and were used to calculate moult durations τ_*n*_ for codes *n* = 1, 2, 3 as follows:

1 $${{\rm{\tau }}}_{n}=(\frac{Firs{t}_{n+1}+Las{t}_{n}}{2})-(\frac{Firs{t}_{n}+Las{t}_{n-1}}{2})$$

where *First* is the first sighting in a given moult code *n*, and *Last* is the last sighting in a given moult code *n*. For instance, the moult stage 1 duration *τ*_1_ is the difference between [the temporal midpoint of the first code 2 sighting and the last code 1 sighting] and [the temporal midpoint of the first code 1 sighting and the last code 0 sighting during the Jan/Feb moult season]. Stage durations were calculated for all seals that were observed in three consecutive moult codes (e.g. codes 0, 1, 2 in the case of *τ*_*1*_), and the distributions of those durations was tested for normality using Lilliefors tests. Moult stage durations did not differ across years, sexes, or reproductive histories (unpaired t-test, p > 0.05 for each stage); thus, data were combined to calculate the mean and standard deviation τ_*n*_ for the duration of each stage. Total moult duration *Τ* was then calculated by summing *τ*_*1*_, *τ*_2_, and *τ*_3_ (Fig. 3). We acknowledge that the existence of a negative co-variance between the duration of τ_n_ and τ_n+1_ results in a conservative estimate of *Τ*. Moult duration values are presented as mean *μ* ± standard deviation *σ* (see Supplemental Materials for *σ* equations).

#### Estimating moult initiation dates

Of the 4252 unique seal-year combinations that were observed during the study, 1208 were observed in both moult codes 0 and 1 (i.e., beginning of moult was known to occur between two set dates. For these individuals, we estimated the moult initiation date as the temporal midpoint between the last code 0 sighting and the first code 1 sighting). Of the remaining individuals, 681 were first observed in moult code 1, 681 in moult code 2, 444 in moult code 3, and 749 in moult code 4. To include the animals in our moult phenology analysis that had not been observed at moult initiation, we back-calculated moult initiation dates for each remaining animal based on their moult code *k* at first sighting *First*_*k*_. Estimating the beginning of a stage required that we first estimate the mean difference Δ_*n*_ between [the midpoint of the first code *n* + 1 sighting and the last code *n* sighting] and [the subsequent code *n* sighting] (Fig. 3), using:

2 $${{\rm{\Delta }}}_{n}=Firs{t}_{n}-(\frac{Firs{t}_{n+1}+\,Las{t}_{n}}{2})$$

for all seals that were observed in two consecutive moult codes (e.g. codes 0, 1 to calculate Δ_1_). Using a Lilliefors test, the Δ_*n*_ distributions were found to be normal. This resulted in average difference Δ_*n*_ values of 5.4 ± 3.9 days (*n* = 347 animals) for Δ_1_, 4.5 ± 2.9 days (*n* = 347 animals) for Δ_2_, 4.3 ± 2.9 days (*n* = 226 animals) for Δ_3_, 5.1 ± 3.7 days (*n* = 213 animals) for Δ_4_ (Table 3). Finally, to back-calculate an initiation date for each animal based on their moult code *k* at first sighting *First*_*k*_, we subtracted the difference value Δ_*k*_ and the sum of the stage durations *τ* for each moult stage *n* in which the animal was not observed:

3 $$InitiationDate=Firs{t}_{k}-{{\rm{\Delta }}}_{k}-\sum _{n=1}^{k-1}{\tau }_{n}$$

**Table 3:** Parameter values used to estimate moult duration and moult initiation dates for all ages and sexes combined.

Parameter	Moult duration (mean μ ± SD *σ)*	Number of Individuals
**τ**_***n***_**;** duration of moult stage *n*		
τ_1_	10.2 ± 5.3 days	70
τ_2_	9.4 ± 4.0 days	73
τ_3_	9.6 ± 3.8 days	50
***Τ*;** duration of entire moult		
	29.2 ± 7.7 days	
**Δ**_***n***_**;** difference between first sighting at stage *n* and previous midpoint		
Δ_1_	5.4 ± 3.9 days	347
Δ_2_	4.5 ± 2.9 days	347
Δ_3_	4.3 ± 2.9 days	226
Δ_4_	5.1 ± 3.7 days	213

To control for inter-annual variation in moult timing, individuals were assigned to moult categories based on the moult initiation date relative to the initiation dates of the other animals in the year of sighting::

- “Early-Moulters”, who initiated moulting before the 25^th^ percentile of the moult initiation dates;
- “Middle-Moulters”, who initiated moulting between (or on) the 25^th^ and 75^th^ percentile of the moult initiation dates; or
- “Late-Moulters”, who initiated moult after the 75^th^ percentile of the moult initiation dates.

### Drivers of moult phenology

To evaluate relationships between moult phenology and sex, year, and reproductive category, we constructed biologically plausible models and then selected the best models using an information-theoretic approach (Table S4). Mixed-effects models were constructed using the package *lme4* and selected using AIC^44,45^ in *R* (R Core Team 2017). The global model was Date_init~Repro_cat*Year*Age + *(1|ID)* where *1|ID* is the random effect of individual and *Repro_cat* is a combined sex/reproductive history category that includes males and females. Age differed by reproductive category (mean ages for Juvenile Females = 4.45 years old (yo), Males = 8.73 yo, Attendant Parturients = 14.03 yo, Attendant Non-Parturient = 15.12 yo; ANOVA, Tukey HSD post-hoc, *p* < 0.05 for all, except Attendant Parturients:Attendant Non-Parturient *p* > 0.05). However, the model AIC was higher when Age was included in the global model (Table S4); as a result, all ages within a single reproductive category were combined for the remaining analyses. For parturient females, the relationship between Year 2 pup birth date and Year 1 pup birth date (with and without the added factor of Year 2 moult initiation date) was also examined using a linear mixed-effects model with year as a fixed effect and individual as a random effect using the package *lme4* in *R*. Finally, a multinomial logistic regression of Year 2 reproductive success as a function of Year 1 reproductive category plus Year 1 moult category was examined using the package *mgcv* in R.

### Interactions between pupping success/phenology and moult phenology

For sexually mature females, we calculated three sets of transition probabilities:

1. From all Year 1 pupping categories into each moulting category (Table S1; transition probabilities 26% Early-Moulters, 48% Mid-Moulters, and 26% Late-Moulters).
2. From all Year 1 moulting categories into Year 2 pupping categories (Table S2; expected transition probabilities 24% Attendant Non-Parturient, 16% Attendant Early-Parturients, 32% Attendant Mid-Parturients, 17% Attendant Late-Parturients, 12% Non-Attendant Adult Females)
3. From all from Year 1 pupping categories into Year 2 pupping categories (Table S3; expected transition probabilities 22% Attendant Non-Parturient, 16% Attendant Early-Parturients, 31% Attendant Mid-Parturients, 16% Attendant Late-Parturients, 14% Non-Attendant Adult Females).

These “expected” transition probabilities (null hypothesis; seals transition from one category to another with equal probabilities) were compared against the “observed” transition probabilities using a Markov simulation on 10,000 multinomial draws. P-values were adjusted to account for table-wide Type I errors using a Bonferonni-type correction^46^ (see Supplemental Material).

## Results

### Demography of moulting animals

Survey frequencies and counts for each study year are provided in Table 1. We observed 2% of all animals during all four study years, 11% during three years, 25% during two years, and all other animals during only one study year (62%). Tagged animals (all ages and sexes) were observed an average of 2.1 ± 1.4 times within a moulting season (minimum 1, maximum 15, median 2, mode 1). Of the 4252 seals seen during the moult, 63% of animals had been seen during the lactation period several weeks earlier. For females, the composition of Juvenile Females, Attendant Parturients, and Attendant Non-Parturient seen during the moulting period stayed relatively consistent within and across years, averaging 23%, 51%, and 26%, respectively.

### Moult duration

Moult stage durations were 10.2 ± 5.3 days for *τ*_*1*_ (stage 1), 9.4 ± 4.0 days for *τ*_2_ (stage 2), and 9.6 ± 3.8 days for *τ*_3_ (stage 3) (Table 3). Using these average stage durations, the entire visible moult duration *Τ* was 29.2 ± 7.7 (mean ± standard deviation) days for Weddell seals. Using Equation 5, animals first seen in moult codes 1 (i.e. *k* = 1), 2, 3, and 4 had *σ*_*Date_init*_ values of 3.90 days (*n* = 1007), 6.04 days (*n* = 503), 7.25 days (*n* = 251), and 8.50 days (*n* = 369), respectively. Note that animals with only sightings at code 0 were excluded (*n* = 182) such that the total number of animals assigned initiation dates was 2130.

### Links between pupping phenology and moulting phenology in one season

Moult initiation date ranged from Dec 09 to Feb 28 with a mean start date of January 15 ± 13.5 (SD) days, although the variance was generally smaller within each reproductive category. Based on the lowest AIC value and Akaike weight, the best mixed-effects model included the interaction between *Repro_cat* and *Year* (Table S4). Thus, the wide range (81 days) of moult initiation dates likely resulted from influences of year and reproductive categories.

**Figure 4:**
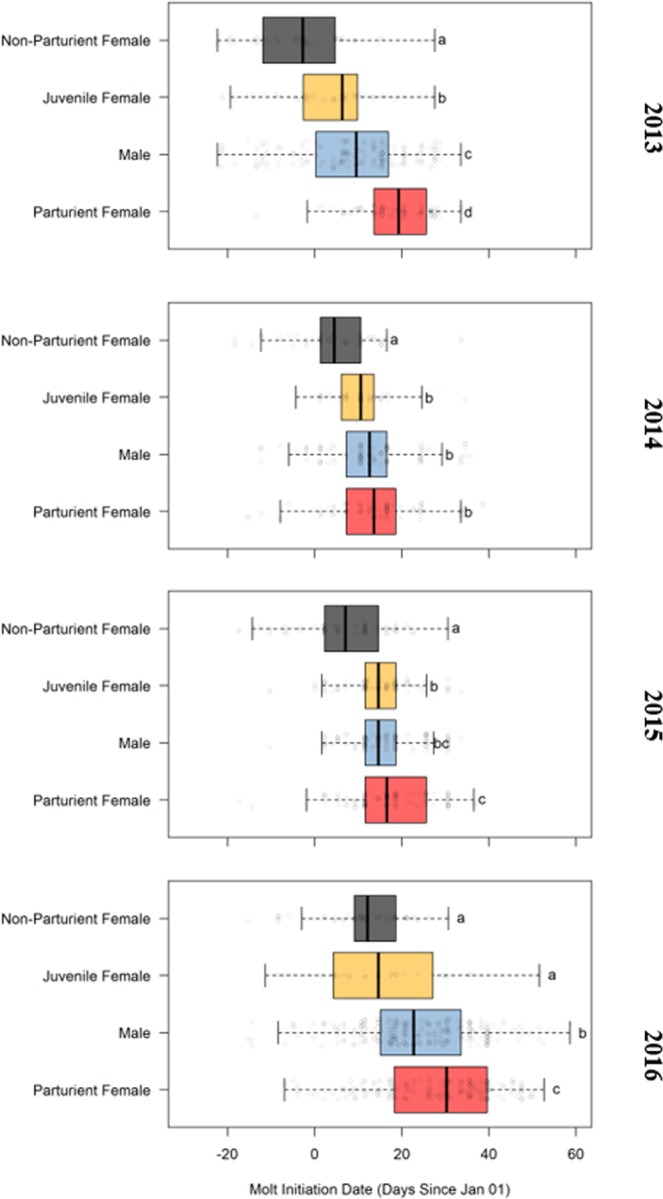
Moult initiation dates across reproductive categories and years (panels; 2013 is the 2013 austral summer including the December 2013 – February 2014 moult) for Attendant individuals. Within each year, different letters denote significantly different moult initiation dates across reproductive categories (Tukey’s HSD, p < 0.05). During all study years, sexually mature females that did not produce a pup (Non-Parturients) moulted earlier than all other reproductive categories. On the contrary, sexually mature females that produced a pup (Parturients) tended to moult later than sexually immature females (Juvenile Females, significant difference in 2013, 2015, 2016), and Males (significant difference in 2013, 2016).

**Figure 5:**
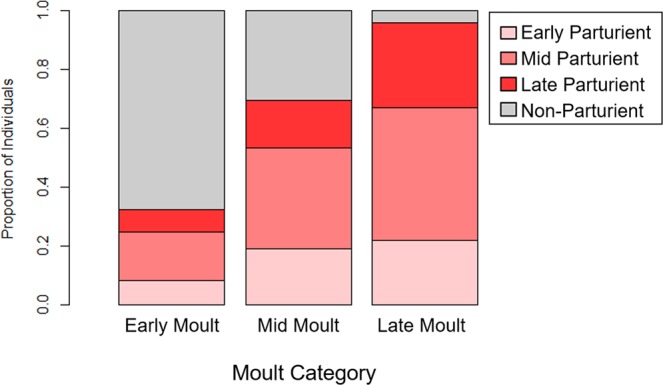
Proportion of seals in each moult category in Year 1 comprised of different reproductive categories from Year 1. Moult phenology was not independent of pupping phenology: the Early-Moulters category was predominated by Attendant Non-Parturient, whereas the Late-Moulters category was predominated by Attendant Parturients.

The resulting transition probabilities from pupping categories to moulting categories in each year are provided in Table S1. Attendant Non-Parturient had the earliest average moult initiation dates (range December 28 to January 12 across study years) followed by Juvenile Females (January 04 to January 15), Males (January 09 to January 24), and females that had given birth (Attendant Parturients; January 15 to January 29) (Fig. 4). Given that eight of fifteen transition outcomes differ significantly from expected, the data strongly suggest that moult phenology is not independent from pupping phenology in a given year (Table S1) (Fig. 5). Specifically, the Early-Moult category is more likely to be comprised of Attendant Non-Parturient (28% greater than expected) than Attendant Early-, Mid-, or Late- Parturients (13%, 13%, and 15% less than expected, respectively). Animals in the Mid-Moult category were disproportionately composed of animals that had been Non-Attendant during the previous pupping period (14% greater than expected). Animals that pupped contributed significantly more than expected to the Late-Moult category, with Attendant Late-Parturients (46%) contributing more than Attendant Mid-Parturients (39%), or Attendant Early-Parturients (36%). For Attendant Parturients, the moult initiation date was significantly related to when the pup was born (linear mixed effects model, R^2^ = 0.24). Thus, moult phenology is delayed in Attendant Parturients relative to Attendant Non-Parturients, and there is a direct relationship between date of pupping and moult onset.

### Inter-annual variation in ice dynamics, moult phenology, and colony attendance

Among study years, the moult was earliest in 2013 (mean moult initiation date January 08 ± 13 days) and latest in 2016 (January 22 ± 14 days) with moult onset during the two intermediate years occurring in between (January 11 ± 11 days in 2014 and January 14 ± 10 days in 2015) (Fig. 4, Table 1). These inter-annual differences in moult initiation date were supported by the raw survey data. In the first survey of 2013, 33% of observed seals had yet to begin moulting (moult code 0) and 12% had already completed the moult (moult code 4), whereas in a 2016 survey on that same date, 47% of seals had yet to begin moulting, and only 1% had completed the moult. The one exception was Attendant Parturients, in which moult initiation began significantly earlier in 2014 (January 15) than 2013 (January 19) and 2014 (January 18) (Fig. 4, Table 1, TukeyHSD on ANOVA, *p* < 0.05). While the 2016 moult surveys extended later than other years (Table 1), this would not have impacted moult initiation dates, as most seals seen after February 13–15 (final survey dates of 2013, 2014, and 2015) had started to moult (and thus moult start date would have already been detected prior to the end of surveys). Indeed, removing the 2016 sightings after February 15 still resulted in significantly later 2016 moult start dates as compared to other years for all reproductive categories. Sea ice break-out date varied by 33 days across the study years and both ice break-out and moult initiation dates were later in 2016 than other years (Tale 1). The within-year moult phenology variance in our study may be artificially high because we do not control for some factors known to affect moult start (e.g., circulating hormone concentrations^47^, body condition^36^).

### Links between pupping and moulting phenology in one season and pupping in the next

For sexually mature females, Year 2 pupping categories (Pupping, Skip-Pupping, or Non-Attendant) were significantly related to moult initiation date in Year 1 (chi-square test, χ^2^ = 18.923, *p* = 0.0153): Early-Moulters contributed 4% less than expected to the Non-Attendant-Parous category whereas Late-Moulters showed the opposite trend, contributing 6% more than expected to Non-Attendant-Parous (Table S2). However, results also indicate that moult phenology reflects previous reproduction dynamics rather than driving future reproduction dynamics, and that the Year 2 pupping categories were more strongly related to the Year 1 pupping categories, than by the dates of the intervening moult (chi-square test, χ^2^ = 130.52, *p* < 0.0001, Table S3). Further, Year 1 moult timing did not help explain whether animals became Parturient, Non-Parturient, or Non-Attendant in Year 2 (multinomial logistic regression, AIC without moult = 1339, AIC with additive effect of moult = 1340). In general, individuals in a given pupping category in Year 1 were likely to remain in the same reproductive phenology category in Year 2 (Table S3) and there was a strong relationship between Y1 and Y2 birthdates (*Y2PupDate* = *0.63(*±SE 0.06)*Y1PupDate-23.64, R^*2*^ = *0.292*). When added into a multiple regression, moult timing was not a significant explanatory factor in Y2 birthdates (*P* = 0.173). Thus, contrary to initial expectations, Year 2 pupping success and phenology are driven by Year 1 reproductive dynamics rather than the intervening moult phenology.

## Discussion

### Reproductive history affects moult phenology

Within sexually mature individuals, we found that post-parturient females and males moulted later than non-parturient females. Within parturient females, we found that later birth was associated with later moult (see Table S1). Moult initiation is likely delayed in these groups relative to sexually immature and non-parturient individuals by the elevated circulating cortisol^48^ and prolactin^49^ levels during lactation and elevated testosterone during breeding^47,50^. Energetics may also contribute to carryover effects between reproduction and moult; specifically, reduced body condition following offspring care may delay moult onset until individuals regain enough energy stores by foraging. A similar phenomenon has been noted in several species of seals^33–37,51^, birds^13,14,24–27^, and terrestrial mammals^52–54^.

The energetic implications of late moult onset in parturient individuals is unclear. Because epidermal cells have a minimum temperature threshold for mitotic division^55^, mismatches between moult phenology and ambient conditions could lead to higher temperature differentials and consequently higher heat loss. In our study, the moult initiation date of Attendant Non-Parturient (January 06 ± 12 days) aligned with the warmest air temperatures of the year (January 03–05^56^); in contrast, Attendant Parturients initiated moult on average 16 days later (in up to 5 °C colder temperatures) than Attendant Non-Parturients (see Table 1). Thus, by moulting later, parturient individuals may experience increased moult costs and require additional prey resources. These costs, in addition to the high energetic costs of lactation^57^, may result in Parturients beginning the next reproductive cycle in poorer body condition, which could in turn lower pup weaning mass^58^ and diminish post-weaning survival and recruitment^59^. However, higher quality individuals may be able to make up for these additional costs by foraging more over the intervening winter and spring^60,61^.

### Inter-annual variation in ice dynamics affects moult phenology

We found a significant effect of year on moult initiation dates, with moult beginning earliest in 2013 and latest in 2016 (see Table 1, Fig. 4) in most reproductive categories. The marked inter-annual differences in moulting phenology across 2013 and 2016 was reflective of sea ice break-out phenology: in 2016, the McMurdo Sound ice break-out occurred 21 days later^62^ and the moult occurred 10–15 days later than in 2013. Limited pack ice retreat has been found to stunt and delay the annual phytoplankton bloom^63^ which would impact the food resources of Weddell seals^64^. Low resource availability and consequently poor body condition may delay moult via increased cortisol levels and suppressed thyroid hormones^28,65^ as has been found in birds^8^.

### Cross-year carryover effects between moulting and pupping

While there were links between Year 1 moult and Year 2 pupping, our data suggest Year 2 reproductive success is driven primarily by Year 1 pupping success and phenology rather than Year 1 moulting phenology (Tables S2, S3). In general, parous seals were likely to remain in the same pupping categories across Years 1 and 2 (Table S3); however, we found that Attendant Late-Parturients were more likely than expected to become Non-Attendant the following year. It is common for sexually mature birds and mammals to intermittently skip reproduction^66^ because it takes individuals more than one calendar year to acquire the capital needed for future reproduction^67^. In support of this mechanism, non-breeding individuals are often in poorer quality due to stress, starvation, diseases, or parasites^68^. We suggest that energetic constraints may be responsible for the increased probability of Attendant Late-Parturients becoming Non-Attendant: individuals with lower energy reserves are commensurately less likely to attend breeding colonies^69^. Similar effects have been seen in other species. In red voles, for example, females that successfully reproduce and consequently moult later have lower overwinter survival due to delayed winter preparation^53,70^.

We found no effect of sex, reproductive category, or year on moult duration, which we estimated to be 29.2 ± 7.7 (mean ± SD) days in Weddell seals. This moult duration is similar to non-catastrophic moult durations in other phocid seals and notably shorter than those of fur seals and sea lions (Family *Otariidae*; Table S5). Recent evidence suggests that Weddell seal life history events fill nearly an entire year, with embryonic diapause being very short or non-existent^71^, gestation lasting 10 months^72^, visible moult lasting 29 days (this study), and lactation lasting 45 days^73^; however, some Weddell seals produce pups in many sequential years^74^ so a >365 day life history cycle is unlikely, at least for the best performing individuals.

In our study, Non-Attendant individuals were 10% more likely than expected to remain Non-Attendant (Table S3), although 76% of individuals returned to breeding colonies the subsequent year. Temporary emigration can reduce conspecific conflict and food competition but precludes breeding opportunities and may increase predation risk because there is more open water access for predators^75^. We found that the probability of colony attendance fluctuated across years, with more Non-Attendants and less Attendant Parturients following years of early (2015) or late (2016) ice break-out relative to years with more typical ice break-out phenology (Table 1). In juvenile Weddell seals, increased sea ice extent has been found to result in more frequent emigration, probably because higher sea ice extent corresponds to lower primary production and presumed lower foraging success^69^, which in turn lowers the number of individuals able to reach the body condition threshold necessary for attending colonies^76^. Another explanation is a shift in age structure following highly productive years^77^ to a higher frequency of older females, which are more likely to become Non-Parturient^78^ and Non-Attendant^39^. In our study, inter-annual variation in colony attendance is likely an interaction between shifts in population age structure and fluctuating environmental conditions.

### Implications of phenology disruptions

Phenological disruptions are increasingly likely under predicted global change scenarios^79^ and have already been documented in several species. For instance, breeding phenology advancement has been associated with spring temperature increases^80–83^. Phenology disruptions may carry-over to other life history events or other years^84^, and have larger impacts on population health than predicted if treated in isolation. These carry-over effects are particularly concerning in high-latitude environments that have stronger selection pressures^85,86^. Furthermore, species may differ in their phenological plasticity, which can lead to mismatches between interacting species such as predators and prey.

To fully understand the ecological impacts of changing environments, researchers must first characterize the full annual cycle of life history events and how they interact with each other physiologically. Unlike Weddell seal birth phenology that is consistent across years and individuals (range = 37 days, SD = 7 days,^74,87^), we found that moult timing is much more flexible (range = 75 days, SD = 14 days) without compromising reproductive success or altering future reproductive phenology in subsequent years. Thus, our data provide encouraging evidence that Weddell seals have some inherent phenological flexibility within the annual cycle with which to respond to fluctuating environmental conditions.

## Supplementary information

Supplementary Information

## Data Availability

Data are available at: http://www.usap-dc.org/view/dataset/601131.
