# Supplementary material for: Reproductive success delays moult phenology in a polar mammal
Source: Sci Rep. 2019 Mar 26;9:5221. doi: 10.1038/s41598-019-41635-x (PMC6435649; doi:10.1038/s41598-019-41635-x)
Supplement: Supplementary file 1 — Supplementary Information [file 41598_2019_41635_MOESM1_ESM.docx]

**Supporting Information**

Reproductive success delays moult phenology in a polar mammal

Roxanne S Beltran^1,2,3,*^, Amy L Kirkham^2,4^, Greg A Breed^5^, J Ward Testa^2,6^, Jennifer M Burns^2^

^1^ Biology and Wildlife, University of Alaska Fairbanks, 2090 Koyukuk Drive, Fairbanks, Alaska 99775, USA

^2^ Biological Sciences, University of Alaska Anchorage, 3101 Science Circle, Anchorage, Alaska 99508, USA

^3^ Current address: Ecology and Evolutionary Biology, University of California Santa Cruz, 115 McAllister Way, Santa Cruz, California 95060, USA

^4^ College of Fisheries and Ocean Sciences, University of Alaska Fairbanks, 17101 Point Lena Loop Road, Juneau, Alaska 99801

^5^ Institute of Arctic Biology, University of Alaska Fairbanks, P.O. Box 757000, Fairbanks, Alaska 99775, USA

^6^ Marine Mammal Laboratory, Alaska Fisheries Science Centre, National Marine Fisheries Service, National Oceanic and Atmospheric Administration, 7600 Sand Point Way N.E. F/AKC3, Seattle, W 98115, USA

^*^ Corresponding author, [roxanne.beltran@gmail.com](mailto:roxanne.beltran@gmail.com), ORCID 0000-0002-8520-1105

**Fieldwork Methods**

Inclement weather and logistical constraints occasionally reduced visitation frequency. For the analyses presented here, we only used data from pups whose birth dates could be precisely determined based on appearance of the pup, placenta, and/or umbilical stump (1). In 2013, the United States government shutdown delayed the start of annual pup tagging efforts until October 29, typically the median of the pupping distribution (Table 1). As a result, pup birthdates are more uncertain in 2013 for pups born before October 29. To estimate birth dates for 2013 with the highest accuracy possible, we estimated birthdates based on the visual presence of an umbilical cord on each pup: the birthdate was considered six days prior to first sighting if there was no umbilical cord, four days prior to sighting if there was an umbilical cord but the pup was estimated to be older than two days based on size, or one day prior to sighting if the pup was noted as new-born. Because the number of pups born in 2013 (*n*=544) was known, we assumed that the pupping distribution was normally distributed as in other years (1). Thus, we assigned the median date when half the pups had been born (272^nd^ pup; October 29^th^) and the 75^th^ percentile when three quarters of the pups had been born (408^th^ pup; November 02^nd^; (1)). Due to the uncertainty in pupping dates prior to the median, we calculated the categories for 2013 parturient females as follows: Attendant Early-Parturients, who gave birth before the 50^th^ percentile of the pupping distribution (32% of individuals); Attendant Mid-Parturients, who gave birth between (or on) the 50^th^ and 75^th^ percentile of the pupping distribution (48%); and Attendant Late-Parturients, who gave birth after the 75^th^ percentile of the pupping distribution (20%).

**Analytical Methods**

Encounter histories for each animal were sorted by date and filtered to exclude repeated observations of the same animal on the same day, or cases in which moult code at time [t] was not less than or equal to the moult code at time [t+1] (i.e. the moult progression was biologically impossible). This led to the removal of 5.5% of sightings.

To account for the propagation of error resulting from summing *τ_n_* components with separate standard deviation terms, we calculated an overall moult duration standard deviation *σ_Τ_* using the following equation:

$$\sigma_{T}=\sqrt{\sum_{n=0}^{k} \left( \sigma_{\tau_{n}} \right)^{2}}$$

Similarly, we calculated a cumulative error value for the moult initiation date of each seal as follows:

$$\sigma_{Date_{init}}=\sqrt{\left( \sigma_{\Delta_{k}} \right)^{2}+\sum_{n=0}^{k} \left( \sigma_{\tau_{n}} \right)^{2}}$$

where ***σ***_τn_ is the standard deviation of moult duration for moult code *n,* summed from moult code 0 (*n*=0) to the moult code *k* at first sighting (*n*=*k*). In this way, the extrapolated moult start date for animals that were first sighted in moult code 4 would have a larger estimated error than for animals that were first sighted in moult code 1.

For the transition probability matrices, a Bonferonni-type correction was used to account for table-wide Type I errors. An adjusted test-wise critical value *αA* was calculated for each cell using *αA* =*αPF*/C where *αPF* is the family-wise critical value of 0.05 and *C* is the number of significant cells. Unadjusted *αU* values were ranked from smallest to largest and evaluated against *αA* values corrected for that many cells (*C*=*n*). If the unadjusted *αU* value was still less than the adjusted *αA* value, it was retained (*** in Tables 4-6); otherwise it was considered non-significant. The next highest unadjusted *αU* value was then compared to the next adjusted *αA* for *C=n-1* cells, and so on for each subsequent *αU* value.

NCEP Reanalysis air temperature data was provided by the NOAA/OAR/ESRL PSD, Boulder, Colorado, USA. Ice break-out date from the US National Snow and Ice Data Center NASA Bootstrap SMMR-SSM/I combined dataset.

**Table S1.** Transition probabilities from pupping categories in the Year 1 pupping season (rows) and moulting categories in the Year 1 moulting season (columns) for previously parous females. The expected moult category outcomes are provided in the column headers and assume a random distribution of moult timing across pupping categories, calculated from the contribution of all pupping categories to each moulting category. Each cell contains the number of individuals with that transition outcome (top value), the actual proportion of animals with that transition outcome (middle top value), the actual minus expected outcome (middle bottom value, green if positive, red if negative) and the p-value for the Markov simulation of actual versus expected outcome (bottom value, *** *p*<0.05 with Bonferroni correction). Actual transition outcomes sum to 100% for each reproductive history (row).

|  |  | Moult Year 1 | | |
| --- | --- | --- | --- | --- |
|  |  | Early-Moulter  (expected 26%) | Mid-Moulter  (expected 48%) | Late-Moulter  (expected 26%) |
| Pupping Year 1 | Attendant Non-Parturient | 144  54%  +28%  *** | 114  43%  -5% | 9  3%  -23%  *** |
|  | Attendant Early-Parturient | 18  13%  -13%  *** | 72  51%  +4% | 50  36%  +9% |
|  | Attendant Mid-Parturient | 35  13%  -13%  *** | 129  48%  +1% | 103  39%  +12%  *** |
|  | Attendant Late-Parturient | 16  11%  -15%  *** | 62  43%  -5% | 66  46%  +20%  *** |
|  | Non-Attendant Adult Females | 23  27%  +1% | 53  62%  +14% | 10  12%  -15%  *** |

**Table S2.** Transition probabilities of parous female Weddell seals from Year 1 moulting categories (rows) to Year 2 pupping categories (columns). See Table S1 legend for description of values.

|  |  | Pupping Year 2 | | | | |
| --- | --- | --- | --- | --- | --- | --- |
|  |  | Attendant Non-Parturient  (expected 24%) | Attendant Early-Parturient  (expected 16%) | Attendant Mid-Parturient  (expected 32%) | Attendant Late-Parturient  (expected 17%) | Non-Attendant Adult Females  (expected 12%) |
| Moult Year 1 | Early-Moulter | 82  28%  +5% | 53  18%  +3% | 87  30%  -2% | 45  16%  -1% | 23  8%  -4%  *** |
|  | Mid-Moulter | 118  23%  -1% | 70  14%  -2% | 169  33%  +1% | 90  18%  +1% | 61  12%  0% |
|  | Late-Moulter | 48  19%  -4% | 39  16%  0% | 76  31%  -1% | 40  16%  -1% | 44  18%  +6%  *** |

**Table S3.** Transition probability from pupping Year 1 to pupping Year 2 (ignoring the intermediate moult phenology). See Table S1 legend for description of values.

|  |  | Pupping Year 2 | | | | |
| --- | --- | --- | --- | --- | --- | --- |
|  |  | Attendant Non-Parturient  (expected 22%) | Attendant Early-Parturient  (expected 16%) | Attendant Mid-Parturient  (expected 31%) | Attendant Late-Parturient  (expected 16%) | Non-Attendant  Parous  (expected 14%) |
| Pupping Year 1 | Attendant Non-Parturient | 72  27%  +5% | 56  21%  +4% | 81  30%  -1% | 31  12%  -5% | 27  10%  -4% |
|  | Attendant Early- Parturient | 37  27%  +5% | 43  31%  +14%  *** | 41  29%  -2% | 7  5%  -11%  *** | 11  8%  -6% |
|  | Attendant Mid- Parturient | 47  18%  -4% | 32  12%  -4% | 103  39%  +8%  *** | 45  17%  +1% | 36  14%  -1% |
|  | Attendant Late- Parturient | 22  15%  -6% | 4  3%  -14%  *** | 32  22%  -9% | 52  36%  +20%  *** | 33  23%  +9%  *** |
|  | Non-Attendant Adult Females | 16  19%  -3% | 13  15%  -1% | 24  28%  -3% | 12  14%  -2% | 21  24%  +10% |

**Table S4.** Model selection for moult initiation date. The Repro_cat*Year interaction includes the two main effects and the interaction.

| **Model** | **K** | **AIC** | **ΔAIC** | **AIC Weight** |
| --- | --- | --- | --- | --- |
| Repro_cat*Year | 5 | 16312.7 | 0 | 1 |
| Repro_cat+Year | 4 | 16392.78 | 80.08 | 4.08E-18 |
| Repro_cat*Year*Age | 7 | 16514.2 | 121.42 | 1.75E-44 |
| Year | 3 | 16719.9 | 407.2 | 3.78E-89 |
| Repro_cat | 3 | 16815.74 | 503.04 | 5.8E-110 |
| Intercept | 2 | 17134.43 | 821.73 | 3.7E-179 |

**Table S5.** Summary of moult durations for pinniped species.

| **Species** | **Family** | **Moult Duration (Days)** | **Moult Type** | **Citation** |
| --- | --- | --- | --- | --- |
| Hawaiian monk seal | *Phocidae* | 8-9 | Catastrophic | (2) |
| Southern elephant seal | *Phocidae* | 7-14 | Catastrophic | (3) |
| Mediterranean monk seal | *Phocidae* | 14 | Catastrophic | (4) |
| Weddell seal | *Phocidae* | 29*±*8 | Gradual | This study |
| Harbor seal | *Phocidae* | 33-35 | Gradual | (5, 6) |
| New Zealand sea lion | *Otariidae* | 60 | Gradual | (7) |
| Northern fur seal | *Otariidae* | 105 | Gradual | (8) |

**Literature Cited**

1. Rotella JJ, Paterson JT, & Garrott RA (2016) Birth dates vary with fixed and dynamic maternal features, offspring sex, and extreme climatic events in a high‐latitude marine mammal. *Ecology and Evolution* 6(7):1930-1941.

2. Johanos TC, Becker BL, & Ragen TJ (1994) Annual reproductive cycle of the female Hawaiian monk seal (*Monachus schauinslandi*). *Marine Mammal Science* 10(1):13-30.

3. Boyd I, Arnbom T, & Fedak M (1993) Water flux, body composition, and metabolic rate during molt in female southern elephant seals (*Mirounga leonina*). *Physiological Zoology*:43-60.

4. Badosa E, Pastor T, Gazo M, & Aguilar A (2006) Moult in the Mediterranean monk seal from Cap Blanc, western Sahara. *African Zoology* 41(2):183-192.

5. Scheffer VB & Slipp JW (1944) The harbor seal in Washington State. *The American Midland Naturalist* 32(2):373-416.

6. Thompson P & Rothery P (1987) Age and sex differences in the timing of moult in the common seal, *Phoca vitulina*. *Journal of Zoology* 212(4):597-603.

7. McConkey S, Lalas C, & Dawson S (2002) Moult and changes in body shape and pelage in known‐age male New Zealand sea lions (*Phocarctos hookeri*). *New Zealand Journal of Zoology* 29(1):53-61.

8. Scheffer VB & Johnson AM (1963) *Molt in the northern fur seal* (US Department of Interior, Fish and Wildlife Service).
